# TOPK and PTEN participate in CHFR mediated mitotic checkpoint^☆^

**DOI:** 10.1016/j.cellsig.2013.08.013

**Published:** 2013-12

**Authors:** Swapnil R. Shinde, Narmadha Reddy Gangula, Sridhar Kavela, Vimal Pandey, Subbareddy Maddika

**Affiliations:** Laboratory of Cell Death & Cell Survival, Centre for DNA Fingerprinting and Diagnostics (CDFD), Nampally, Hyderabad 500001, India

**Keywords:** Chfr, PTEN, TOPK, E3 ligase, Akt, Mitosis

## Abstract

Mitotic progression is regulated by co-ordinated action of several proteins and is crucial for the maintenance of genomic stability. CHFR (*C*heck point protein with *F*HA and *R*ING domains) is an E3 ubiquitin ligase and a checkpoint protein that regulates entry into mitosis. But the molecular players involved in CHFR mediated mitotic checkpoint are not completely understood. In this study, we identified TOPK/PBK, a serine/threonine kinase and PTEN, a lipid phosphatase to play an important role in CHFR mediated mitotic transitions. We demonstrated that CHFR ubiquitinates and regulates TOPK levels, which is essential for its checkpoint function. Moreover, TOPK phosphorylates and inactivates PTEN, which in turn activates Akt that leads to proper G2/M progression. Collectively, our results reveal TOPK and PTEN as new players in CHFR mediated mitotic checkpoint.

## Highlights

• TOPK is identified as a novel CHFR associated protein.
• TOPK is a substrate of CHFR.
• TOPK participates in CHFR mediated mitotic stress check point.
• PTEN is phosphorylated by TOPK and is required for mitotic entry.

## 1 Introduction

CHFR is a well defined mitotic checkpoint protein that delays entry into mitosis in response to stress [1]. The role of CHFR in mitotic checkpoint is attributed to its function as an E3 ligase, which ubiquitinates and degrades its target proteins. Currently CHFR is known to regulate the stability of very limited substrates such as Aurora A, Plk1, Kif22 and PARP1, which are critical for proper mitotic transitions. In addition to its proteosomal substrates, CHFR was also shown to regulate checkpoint in a proteosomal independent manner [2]. For instance ubiquitylation-dependent activation of p38 kinase but not proteasome dependent degradation by CHFR contributes to its checkpoint [3]. Also CHFR controls mitotic transitions by regulating the nuclear localization of cyclin B [4].

So far three modes of regulation have been reported to control CHFR mitotic function. Firstly, PML bodies were shown to control the distribution, dynamics and mitotic checkpoint function of CHFR [5]. Secondly Akt phosphorylates CHFR and may promote its autoubiquitination activity and thus inhibits its checkpoint function [6]. Finally, polyADP-ribosylated substrates catalyzed by PARP1 bind to CHFR *via* its PBZ-motif and thus may be required for its function in ante-phase checkpoint [7].

To elucidate potential substrates and/or regulators of CHFR during its mitotic checkpoint function, we recently performed a tandem affinity purification using a 293 T cell line stably expressing the triple tagged (S-protein, FLAG, and Streptavidine-binding peptide) version of CHFR (SFB-CHFR) and identified several CHFR associated proteins by mass spectrometric analysis [8]. We repeatedly found TOPK as one of the potential CHFR associated proteins. TOPK (lymphokine-activated killer T-cell-originated protein kinase) also known as PBK (PDZ-binding kinase) is a MAPKK-like serine/threonine kinase that was originally identified as a gene that is differentially expressed in IL-2 lymphokine-activated killer T (T-LAK) cells [9]. TOPK is shown to be upregulated during mitosis and its phosphorylation at Threonine 9 residue by cdk1/cyclin B is crucial for its mitotic function [10]. Several studies have indicated that TOPK might be a potential oncogene [11]. Overexpression of TOPK has been reported in various human cancers including lung cancer, breast cancer and lymphomas [12–14]. However, the downstream players in TOPK mediated mitotic progression are poorly understood.

## 2 Results

### 2.1 TOPK is a novel CHFR associated protein

**Fig. 1:**
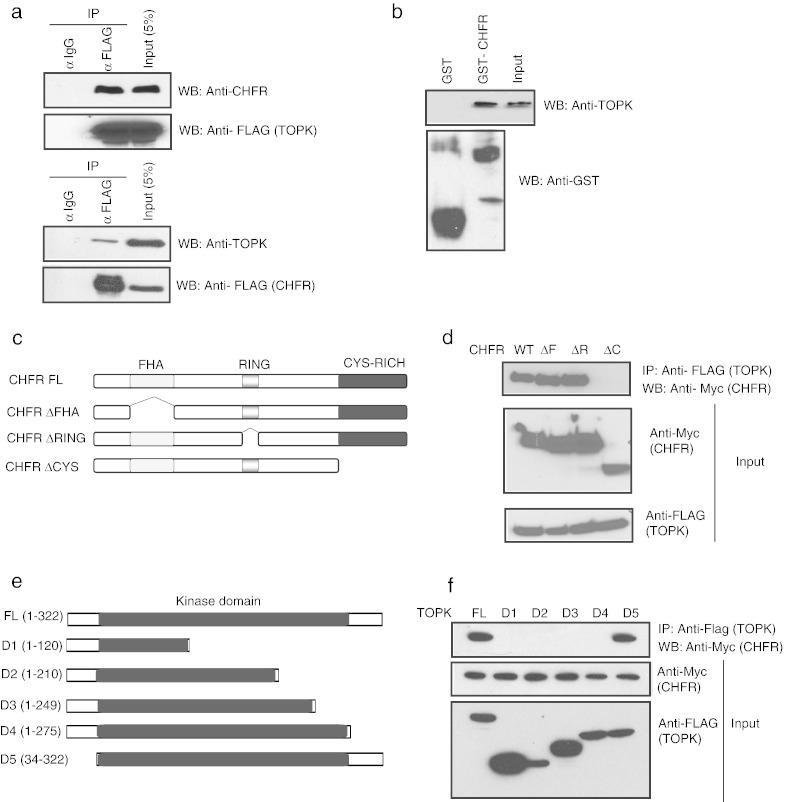
TOPK is a novel CHFR associated protein. (a) 293 T cells were transfected with Flag-tagged TOPK (upper panel) or Flag-tagged CHFR (lower panel). The interaction of CHFR and TOPK was detected by immunoblotting with anti-CHFR and anti-TOPK antibodies after immunoprecipitation with anti-IgG and anti-Flag antibodies. (b) The *in vitro* interaction of endogenous TOPK with CHFR was detected by immunoblotting with anti-TOPK antibody after performing GST pull down assay with either GST or GST–CHFR using 293 T cell lysate. (c) Schematic representation of domain architecture of full length CHFR, along with its various deletion mutants. (d) 293 T cells were co-transfected with the indicated Myc-tagged CHFR constructs along with Flag-tagged TOPK and the interaction was determined by immunoprecipitation and immunoblotting with the indicated antibodies. (e) Schematic representation of N-terminal Flag-tagged full-length TOPK, along with its various deletion mutants. (f) 293 T cells were co-transfected with the indicated Flag-tagged TOPK constructs along with those encoding Myc tagged CHFR and the interaction between TOPK and CHFR was determined by immunoprecipitation and immunoblotting with the indicated antibodies.

We confirmed the association of TOPK with CHFR by co-immunoprecipitation experiments. Endogenous CHFR was found to be associated with exogenously expressed Flag-tagged TOPK in 293 T cells and on the other hand endogenous TOPK was found in the immunoprecipitated CHFR complex (Fig. 1a). Further, we showed that TOPK interacts with bacterially expressed recombinant GST–CHFR but not with GST alone thus confirming the association of TOPK and CHFR (Fig. 1b). In order to map the TOPK binding region on CHFR, we generated full length CHFR along with CHFR deletion mutants that lack various domains (Fig. 1c). Immunoprecipitation data revealed that the TOPK interacted with full length CHFR, ΔFHA, and ΔRING CHFR mutants but not with the Δcysteine rich domain mutant of CHFR (Fig. 1d). Similarly, in order to map the region of interaction of CHFR on TOPK, we generated expression constructs for FLAG–TOPK and a series of deletion mutants that lack various regions of TOPK (Fig. 1e). CHFR interacted with D5 mutant in addition to full length TOPK but not with other mutants suggesting that TOPK region spanning aminoacids 275–322 (C-terminal to kinase domain) is required for CHFR interaction. Together these results suggest that CHFR *via* its C-terminal cysteine rich domain interacts with TOPK at its C-terminal region (Fig. 1f).

### 2.2 TOPK is a substrate of CHFR

**Fig. 2:**
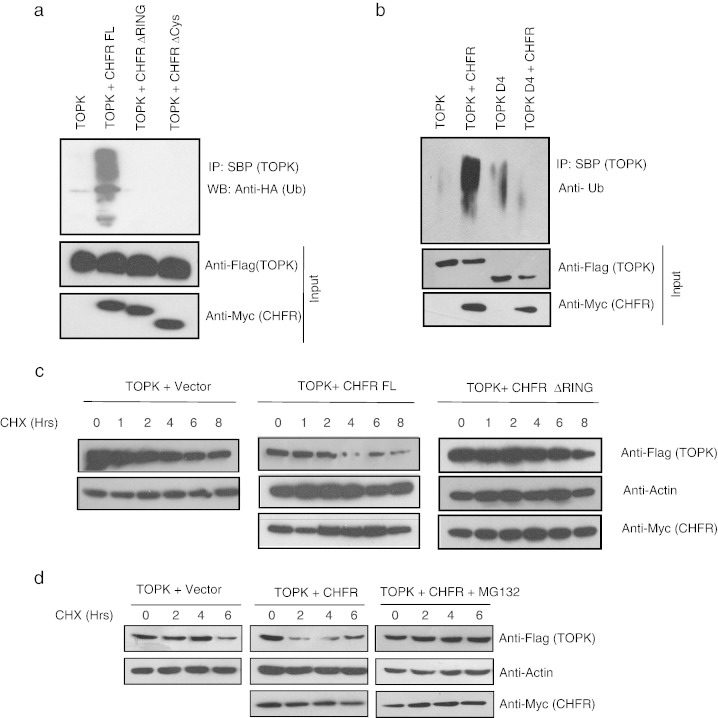
CHFR ubiquitinates and degrades TOPK. (a) Myc tagged full length CHFR, ΔRING or ΔCysteine rich domain CHFR mutants were expressed in HeLa cells along with triple tagged TOPK and HA tagged ubiquitin. After the 24 hour post transfection, cells were treated with MG132 (10 μM) for 6 h and the levels of TOPK ubiquitination were evaluated by immunoprecipitation using streptavidin beads followed by anti-HA immunoblotting. (b) HeLa cells were transiently transfected with TOPK or TOPK D4 constructs along with or without CHFR and TOPK ubiquitination was evaluated by immunoprecipitation using streptavidin beads followed by anti-ubiquitin immunoblotting. (c) HeLa cells transfected with indicated vectors were treated with cyclohexamide and the lysates were collected at the indicated time points. The levels of TOPK, CHFR and actin were determined by immunobloting. (d) HeLa cells were transfected with TOPK along with vector or CHFR and were left untreated or treated with MG132. Cells were treated with cyclohexamide and the lysates were collected at the indicated time points. The levels of TOPK, CHFR and actin were determined by immunobloting.

As TOPK was identified as new associated protein of CHFR, we initially tested if TOPK regulates CHFR function *via* its kinase activity, but we failed to observe any phosphorylation of CHFR by TOPK in a kinase assay. In addition autoubiquitination assays with CHFR either in the presence or absence of TOPK revealed no differences in the E3 ligase activity of CHFR (S.R.S. & S.M., unpublished data), thus may be ruling out the possibility of TOPK as a CHFR regulator. Earlier, CHFR was shown to recruit its substrates such as Aurora A and Kif22 for ubiquitination and degradation *via* its C-terminal cysteine rich domain [8,15]. As TOPK also interacts with CHFR cysteine rich domain we next tested if TOPK is an additional substrate for CHFR E3 ligase activity in cells. Our *in vivo* ubiquitination assays revealed that the polyubiquitination of TOPK occurs only in the presence of full length CHFR but not with the ΔRING or Δcysteine-rich domain deletion mutants (Fig. 2a). On the other hand, full length TOPK but not CHFR binding defective mutant (TOPK D4) was polyubiquitinated by CHFR (Fig. 2b) thus indicating that TOPK might be a substrate of CHFR, which requires intact CHFR E3 ligase activity as well as its interaction domain. Polyubiquitination of TOPK by CHFR is required for its degradation as co-expression of full length CHFR in HeLa cells, which lack endogenous CHFR, reduced protein half-life of TOPK compared to TOPK alone. No change in TOPK half-life was observed with the CHFR ΔRING deletion mutant (Fig. 2c). Further CHFR mediated TOPK degradation was dependent on proteosome activity as pretreatment of cells with a proteosome inhibitor MG132 rescued protein half life of TOPK (Fig. 2d). Together these results suggest that TOPK is an *in vivo* substrate of CHFR E3 ligase activity.

### 2.3 TOPK participates in CHFR mediated mitotic stress check point

**Fig. 3:**
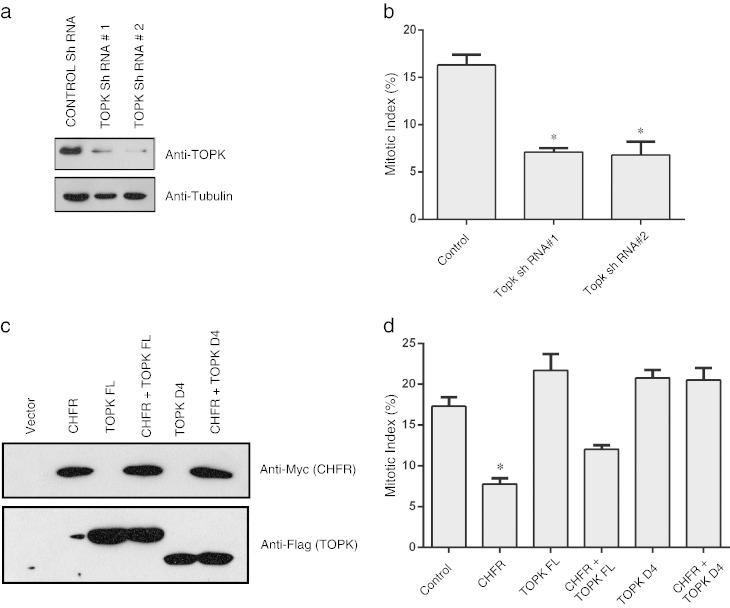
CHFR delays entry into mitosis in TOPK dependent manner. (a) HeLa cells were transfected with either non-targeting control shRNA or two individual TOPK shRNA containing vectors. Cell lysates were collected 48 h later and the expression of TOPK was determined by immunoblotting with TOPK antibody. (b) Cells expressing either control or TOPK shRNAs were treated with Nocodazole for 12 h and later released into a fresh medium. Mitotic index is calculated by scoring phospho-Histone H3 positive cells by immunofluorescence staining after 20 h of Nocodazole release. (c) HeLa cells were transfected as indicated and the expression of the CHFR and TOPK was shown by immunoblotting. (d) HeLa cells were transfected with CHFR and TOPK as indicated and the mitotic index was calculated as in b.

In order to check whether TOPK participates in CHFR induced mitotic check point, we knocked down TOPK in HeLa cells by using shRNAs specifically targeting TOPK (Fig. 3a). Cells were treated with Nocodazole and phospho Histone H3 staining was done after 20 h of Nocodazole release to check the number of cells entering mitosis. Similar to CHFR mediated mitotic stress checkpoint, knock down of TOPK in HeLa cells significantly reduced the number of mitotic cells compared to control cells (Fig. 3b). To further test if CHFR mediated mitotic stress checkpoint is dependent on TOPK we over expressed CHFR alone or in combination with full length TOPK and TOPK D4 mutant in HeLa cells (Fig. 3c) and checked for the phospho Histone H3 positive cells. As expected, overexpression of CHFR delayed cell entry into mitosis whereas TOPK enhanced their entry into mitosis. Interestingly, co-expression of non-degradable TOPK (D4 mutant) along with CHFR significantly rescued CHFR mediated delay into mitosis (Fig. 3d). Together, these results show that TOPK plays an important role in CHFR mediated mitotic check point.

### 2.4 PTEN is phosphorylated by TOPK and is required for mitotic entry

**Fig. 4:**
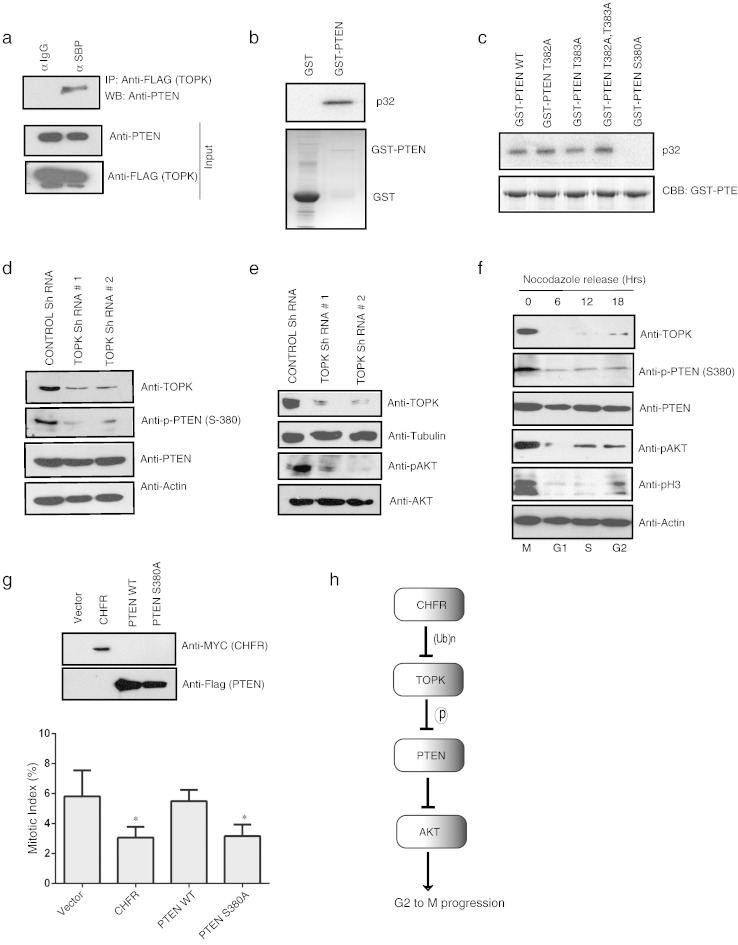
TOPK phosphorylates PTEN and is important for mitotic entry. (a) 293 T cell lysate expressing triple tagged TOPK was used for immunoprecipitation by either anti-IgG sepharose or streptavidine sepharose beads and the interaction of PTEN was detected by immunoblotting with anti-PTEN antibody. (b) An *in vitro* kinase assay was performed on bacterially purified GST or GST–PTEN using a recombinant TOPK holoenzyme in the presence of [γ-p^32^] ATP. Samples were resolved on 10% SDS-PAGE and PTEN phosphorylation was detected by autoradiography. (c) An *in vitro* kinase assay was performed on purified wild type PTEN and various PTEN mutants using an active TOPK enzyme in the presence of [γ-p^32^] ATP and the phosphorylation was shown by autoradiography. (d) PTEN phosphorylation was determined by immunoblotting with phospho-PTEN antibody using HeLa cells transfected with control shRNA or TOPK shRNA containing vectors. (e) Levels of phospho-Akt were determined by immunoblotting with phospho-Akt (serine 473) antibody. (f) HeLa cells synchronized in mitosis by Nocodazole treatment were released into subsequent cell cycle stages. Cell lysates collected at various times were analyzed for the levels of indicated proteins by immunoblotting with their specific antibodies. (g) HeLa cells were transfected as indicated and the expression of the CHFR and PTEN was shown by immunoblotting. Mitotic index is calculated as in Fig. 3b. (h) Proposed model to show the role of TOPK and PTEN in CHFR mediated mitotic checkpoint.

To further understand the mechanism of TOPK participation in CHFR mediated mitotic check point we sought to identify the downstream substrates of TOPK in cells. A recent study has suggested that TOPK promotes AKT activation [13] by reducing cellular PTEN levels and other studies have supported the role of AKT activation in proper G2–M progression [16,17]. Thus we hypothesized that TOPK may participate in CHFR mediated mitotic checkpoint by regulating PTEN–Akt pathway. In an attempt to check whether PTEN is a downstream player in TOPK pathway, first we checked the interaction between TOPK and PTEN. Our immunoprecipitation results indicate that endogenous PTEN interacts with TOPK (Fig. 4a). We next tested whether TOPK can phosphorylate PTEN by using an *in vitro* kinase assay. Autoradiography analysis revealed that GST–PTEN but not GST alone was readily phosphorylated by recombinant TOPK (Fig. 4b). PTEN was reported to be heavily phosphorylated in its tail region (aminoacids 360–390) [18]. In order to map the site of phosphorylation we mutated the serine and threonine residues in this region to alanine. By using various PTEN mutants in a kinase assay we concluded that TOPK phosphorylates PTEN at S380 residue *in vitro* (Fig. 4c). By using a phospho specific antibody we further confirmed PTEN phosphorylation at S380 residue in cells, which is reduced upon TOPK knock down by shRNA (Fig. 4d). In addition, reduced PTEN phosphorylation levels upon TOPK knockdown correlated with decreased Akt activation (Fig. 4e) suggesting that TOPK mediated phosphorylation may lead to PTEN inactivation. This result is in agreement with the previously published work where non-phosphorylatable PTEN mutant was shown to be hyperactive [18], thus suggesting that TOPK might be inactivating PTEN phosphatase by phosphorylating at S380 residue.

To understand the role of PTEN S380 phosphorylation by TOPK in G2 to M progression, we checked the levels of TOPK, p-PTEN (S380), PTEN, and pAkt in different phases of cell cycle. TOPK is highly expressed in mitosis that correlated with high phospho-PTEN (S380) levels and phospho-Akt levels compared to other phases of cell cycle (Fig. 4f). Importantly, phosphorylation of PTEN is required for proper entry into mitosis as expression of non-phosphorylatable PTEN S380A mutant significantly reduced the mitotic cell population (Fig. 4g), which is similar to the observed phenotype upon CHFR expression. Taken together these results suggest that TOPK inactivates PTEN by phosphorylating S380 residue specifically during mitosis, which leads to activation of Akt that is required for proper mitotic progression (Fig. 4h).

## 3 Discussion

In summary, we identified TOPK and PTEN as two novel players in CHFR mediated mitotic checkpoint. We demonstrated that CHFR ubiquitinates and regulates TOPK levels, which is essential for its checkpoint function. Moreover, TOPK phosphorylates and inactivates PTEN, which in turn activates Akt that leads to proper G2 to M progression. Earlier studies have shown that TOPK is upregulated during mitosis and is crucial for proper cell cycle progression during this phase [10], but the molecular mechanism of Akt activation during mitosis is unknown. Here we have shown that PTEN is an important downstream substrate for TOPK function for proper mitotic progression. Previous studies have also shown that PTEN modulates cell cycle progression by regulating phosphatidylinositol 3,4,5-trisphosphate and Akt signaling pathway [19] but the precise mechanism of PTEN activation/inactivation during particular phase of the cell cycle is not known. Further, Akt is transiently activated during G1/S and also in G2/M transition phase [16,20]. Here in our study, we provided evidence to support PTEN inactivation *via* its Serine 380 phosphorylation by TOPK specifically during mitosis, which is followed by Akt activation. Thus precise PTEN inactivation during mitosis is required for proper cell cycle transition as cells expressing non-phosphorylatable and constitutively active PTEN S380A mutant were defective in G2 to M progression.

On the other hand, during G2–M transition CHFR levels were downregulated by its autoubiquitination activity but the question remains how CHFR autoubiquitination activity is stimulated during this particular phase of the cell cycle. Previously it was shown that Akt phosphorylates CHFR and is known to regulate its mitotic checkpoint function [6]. It is possible that downstream of PTEN inactivation, Akt phosphorylates CHFR and stimulates its autoubiquitination activity specifically during G2–M transition. Thus based on these data we speculate that CHFR, TOPK, PTEN and AKT may exist in a feedback loop mechanism to precisely control the mitotic transitions.

Several studies have supported the role of CHFR as a potential tumor suppressor [21,22]. CHFR has been found to be either lost or down regulated or its promoter being hypermethylated in several human cancers including breast, prostate, lung and esophageal cancers. In addition, the role of CHFR as a tumor suppressor has been substantiated by CHFR knock-out mice [15], which are cancer prone and develop spontaneous tumors. In our studies as we found TOPK, an oncogene [11–14] and PTEN, a well known tumor suppressor [23,24] to play an intricate role in CHFR functions we speculate that these two proteins may partially contribute to CHFR tumor suppressor function as well.

## 4 Materials and methods

### 4.1 Plasmids

Full length PTEN, TOPK, CHFR and domain deletion mutants of CHFR and TOPK were cloned into Myc and S-protein/FLAG/SBP (streptavidin binding protein) — triple tagged destination vector using Gateway cloning system (Invitrogen). The point mutants for PTEN were generated by PCR-based site-directed mutagenesis and verified by sequencing.

### 4.2 Antibodies

Anti-PTEN, anti-TOPK, anti-Akt, anti-phospho Akt (Ser 473), anti-phospho PTEN (Ser380) (all from Cell Signalling Technology), anti-Myc clone 9E10, anti-GST (Santa Cruz Biotechnologies), anti-Flag, anti-actin, anti-tubulin, anti-HA (Sigma) and anti-phospho Histone H3 (Ser 10) antibodies were used in this study.

### 4.3 Cell transfections, immunoprecipitation and immunoblotting

Cells were transfected with various plasmids using PEI transfection reagent. For immunoprecipitation assays, the cells were lysed with NETN buffer (20 mM Tris–HCl, pH 8.0, 100 mM NaCl, 1 mM EDTA, 0.5% Nonidet P-40) containing 50 mM β-glycerophosphate, 10 mM NaF, 1 μg/ml of each pepstatin A and aprotinin on ice for 30 min.. The whole cell lysates obtained by centrifugation were incubated with 2 μg of specified antibody bound to either protein A or protein G sepharose beads (Amersham Biosciences) for 1 h at 4 °C. The immunocomplexes were then washed with NETN buffer four times and applied to SDS-PAGE. Immunoblotting was performed following standard protocols.

### 4.4 Retrovirus production and infection

Retroviral vector containing either control shRNA or TOPK shRNA (purchased from Open biosystems) along with a PcL-Ampho helper plasmid was co-transfected in to BOSC23 packing cells. Virus-containing supernatant was collected 48 and 72 h after transfection and was used to infect HeLa cells in the presence of polybrene.

### 4.5 In vitro kinase assay

Bacterially purified GST–PTEN WT, PTEN T382A, PTEN T383A, PTEN 2 T/A (T382A, T383A), PTEN S380A bound to glutathione–Sepharose beads (Amersham) were incubated with kinase assay buffer (25 mM HEPES, 25 mM β-glycerophosphate, 25 mM MgCl2, 2 mM dithiotreitol, and 0.1 mM NaVO3) in the presence of γ-p^32^ labeled ATP with recombinant TOPK holoenzyme for 60 min at 30 º C. The beads were washed and proteins were resolved on 10% SDS-PAGE gel followed by autoradiography analysis.

### 4.6 In vivo ubiquitination assay

HeLa cells were transfected with various combinations of plasmids as indicated in Fig. 2a. After the 24 hour post-transfection, the cells were treated with MG132 (10 μM for 6 h) and the whole cell extracts prepared by NETN lysis were subjected to immunoprecipitation of the substrate protein. The analysis of ubiquitination was performed by immunoblotting with anti-ubiquitin antibodies.

### 4.7 Cell synchronization and mitotic index determination

HeLa cells were treated with the Nocodazole (500 ng/ml) for 12 h, a mitotic shake-off was performed and the cells were reseeded in complete medium for release from the arrest. The analysis of cell cycle was performed different times post Nocodazole release by flow cytometry using propidium iodide staining and expression of proteins were detected by immunoblotting. For mitotic index determination cells collected at 20 hour post release were subjected to immunofluorescence using phospho-Histone H3 antibody using standard protocols. The phospho-H3 positive cells were counted and percentage of mitotic cells was plotted.

## Conflicts of interest

The authors declare that they have no conflict of interest.

## Author contributions

SRS, NRG, VP and SK performed the experiments. SRS, NRG and SM designed the experiments, analyzed the data and wrote the manuscript.
